# Risk of end-stage renal disease in patients with hypertrophic cardiomyopathy: A nationwide population-based cohort study

**DOI:** 10.1038/s41598-019-50993-5

**Published:** 2019-10-10

**Authors:** Heesun Lee, Kyungdo Han, Jun-Bean Park, In-Chang Hwang, Yeonyee E. Yoon, Hyo Eun Park, Su-Yeon Choi, Yong-Jin Kim, Goo-Yeong Cho, Hyung-Kwan Kim, Steve R. Ommen

**Affiliations:** 1 Division of Cardiology, Department of Internal Medicine, Seoul National University College of Medicine, 101 Daehak-ro, Jongno-gu, Seoul, 03080 Korea; 2 Healthcare System Gangnam Center, Seoul National University Hospital, Seoul, Korea; 3 Department of Medical Statistics, College of Medicine, The Catholic University of Korea, Seoul, Korea; 4 Cardiovascular Imaging Section, Cardiovascular Center, Seoul National University Hospital, 101 Daehak-ro, Jongno-gu, Seoul, 03080 Korea; 5 Department of Cardiology, Cardiovascular Center, Seoul National University Bundang Hospital, Seongnam, Gyeonggi South Korea; 6 Division of Cardiovascular Diseases, Mayo Clinic, College of Medicine, Rochester, Minnesota USA

**Keywords:** Cardiac hypertrophy, End-stage renal disease

## Abstract

Although hypertrophic cardiomyopathy (HCM), the most common inherited cardiomyopathy, has mortality rate as low as general population, previous studies have focused on identifying high-risk of sudden cardiac death. Thus, long-term systemic impact of HCM is still unclear. We sought to investigate the association between HCM and end-stage renal disease (ESRD). This was a nationwide population-based cohort study using the National Health Insurance Service database. We investigated incident ESRD during follow-up in 10,300 adult patients with HCM (age 62.1 years, male 67.3%) and 51,500 age-, sex-matched controls. During follow-up (median 2.8 years), ESRD developed in 197 subjects; 111 (1.08%) in the HCM, and 86 (0.17%) in the non-HCM (incidence rate 4.14 vs. 0.60 per 1,000 person-years, *p* < 0.001). In the HCM, the incidence rate for ESRD gradually increased with age, but an initial peak and subsequent plateau in age-specific risk were observed. HCM was a significant predictor for ESRD (unadjusted HR 6.90, 95% CI 5.21–9.15, *p* < 0.001), as comparable to hypertension and diabetes mellitus. Furthermore, after adjusting for all variables showing the association in univariate analysis, HCM itself remained a robust predictor of ESRD development (adjusted HR 3.93, 95% CI 2.82–5.46, *p* < 0.001). The consistent associations between HCM and ESRD were shown in almost all subgroups other than smokers and subjects with a history of stroke. Conclusively, HCM increased the risk of ESRD, regardless of known prognosticators. It provides new insight into worsening renal function in HCM, and active surveillance for renal function should be considered.

## Introduction

Hypertrophic cardiomyopathy (HCM) is the most common genetic cardiomyopathy, with a prevalence of approximately 1:500 in the general population^1,2^. With recent advances in cardiac imaging technologies and genetics, HCM is likely to be detected more frequently than previously thought^3^. HCM is characterized by cardiac hypertrophy without any conditions causing chronic pressure loading on left ventricle (LV)^1^. Despite the common myocardial structural change, clinical manifestations and prognosis of HCM are heterogeneous, ranging from lifelong asymptomatic status to heart failure (HF) or sudden cardiac death^1,4,5^. Until now, clinical interests have focused on identifying patients at higher risk and preventing unfavorable outcomes, particularly sudden death^3–5^. These allow clinicians to understand the disease better and extend survival^1^. Under the current comprehensive management, the majority of HCM patients remain symptom-free throughout their lives, with an annual mortality rate of approximately 1%, as low as general population^5^. Given the low mortality and chronic features of HCM, the long-term influence of HCM on organs other than heart should be noted. Kidney is the representative systemic organ that interacts with the heart. Chronic kidney disease is a strong risk factor for cardiovascular morbidity and mortality, aggravating outcomes proportional to renal dysfunction^6,7^. Among patients with end-stage renal disease (ESRD), approximately 50% of the deaths are caused by cardiovascular disease^8^. Conversely, diverse cardiac diseases, including HF, can deteriorate renal function^6,9^. Since progressive clinical or subclinical HF, attributable to not only LV diastolic but also systolic dysfunction, is one of the most important complications of HCM^5^, renal function can be affected by HCM in the long term. However, the impact of HCM on kidney remains unknown. We sought to investigate the incidence and risk of ESRD development in patients with HCM via a large-scale nationwide cohort study.

## Results

### Baseline characteristics

In the present cohort (n = 61,800; mean age 62.1 years; male 67.3%), HCM patients were more likely to be obese and smokers; had a higher prevalence of comorbidities, such as hypertension, diabetes mellitus (DM), and hypercholesterolemia; more frequent use of renin-angiotensin-aldosterone system (RAS) blocker, beta blocker, calcium-channel blocker, anti-platelet agent, and statin; slightly lower systolic/diastolic blood pressures; and less frequent income lower 20% group. A history of cardiovascular disease, including ischemic heart disease (IHD), stroke, HF, and atrial fibrillation (AF), was more frequent in the HCM than the non-HCM group (all *p* < 0.001). Compared with the non-HCM group, pre-existing renal disease (21.6% vs. 17.2%, *p* < 0.001) and proteinuria (10.1% vs. 5.9%, *p* < 0.001) were observed more frequently, and eGFR (80.7 vs. 87.0 mL/min/1.73 m^2^, *p* < 0.001) was lower in the HCM group (Table 1).

**Table 1:** Baseline characteristics of study population.

	Total (n = 61,800)	HCM group (n = 10,300)	Non-HCM group (n = 51,500)	*p*
***Demographics***				
Age, years	62.1 ± 12.0	62.1 ± 12.0	62.1 ± 12.0	0.999
≥65 years	27,312 (44.2)	4,552 (44.2)	22,760 (44.2)	0.999
Male sex	41,586 (67.3)	6,931 (67.3)	34,655 (67.3)	0.670
Smoking	28,517 (46.1)	4,858 (47.2)	23,659 (45.9)	<0.001
Current smoking	12,864 (20.8)	1,950 (18.9)	10,914 (21.2)	<0.001
BMI, kg/m^2^	24.3 ± 3.1	25.1 ± 3.2	24.1 ± 3.1	<0.001
BMI ≥25 kg/m^2^	23,965 (38.8)	5,096 (49.5)	18,869 (36.6)	<0.001
Systolic BP, mmHg	126.3 ± 15.1	125.4 ± 15.7	126.4 ± 14.9	<0.001
Diastolic BP, mmHg	77.1 ± 9.8	75.8 ± 10.4	77.4 ± 9.7	<0.001
Income lower 20%	11,907 (19.3)	1,864 (18.1)	10,043 (19.5)	0.001
***Previous medical history***				
Hypertension	30,625 (49.6)	7,168 (69.6)	23,457 (45.6)	<0.001
Diabetes mellitus	11,641 (18.8)	2,109 (20.5)	9,532 (18.5)	<0.001
Hypercholesterolemia	21,975 (35.6)	5,201 (50.5)	16,774 (32.6)	<0.001
Ischemic heart disease	13,570 (22.0)	6,033 (58.6)	7,537 (14.6)	<0.001
Heart failure	4,789 (7.8)	2,996 (29.1)	1,793 (3.5)	<0.001
Stroke	4,276 (6.9)	1,137 (11.0)	3,139 (6.1)	<0.001
Atrial fibrillation	2,625 (4.3)	1,756 (17.1)	869 (1.7)	<0.001
Pre-existing renal disease	11,078 (17.9)	2,220 (21.6)	8,858 (17.2)	<0.001
Prior use of anti-platelets	15,525 (25.1)	5,226 (50.7)	10,299 (20.0)	<0.001
Prior use of RAS blocker	19,018 (30.8)	5,075 (49.3)	13,943 (27.1)	<0.001
Prior use of BB	11,893 (19.2)	6,233 (60.5)	5,660 (11.0)	<0.001
Prior use of CCB	18,395 (29.8)	4,718 (45.8)	13,677 (26.6)	<0.001
Prior use of statin	15,572 (25.2)	4,754 (46.2)	10,818 (21.0)	<0.001
***Laboratory findings***				
Hb, g/dL	14.1 ± 1.6	14.3 ± 1.7	14.1 ± 1.6	0.010
Total cholesterol, mg/dL	191.2 ± 38.5	179.3 ± 38.0	193.6 ± 38.2	<0.001
Triglyceride, mg/dL	120.0 ± 8.0	118.0 ± 9.2	120.4 ± 4.4	<0.001
HDL-cholesterol, mg/dL	52.5 ± 14.7	50.9 ± 14.2	52.8 ± 14.7	<0.001
LDL-cholesterol, mg/dL	111.8 ± 37.6	115.4 ± 38.8	113.7 ± 36.8	<0.001
Glucose, mg/dL	104.2 ± 25.9	103.9 ± 24.0	104.2 ± 26.3	0.292
eGFR, mL/min/1.73 m^2^	86.0 ± 47.8	80.7 ± 50.8	87.0 ± 47.1	<0.001
eGFR <60 ml/min/1.73 m^2^	6,174 (10.0)	1,753 (17.0)	4,421 (8.6)	<0.001
Proteinuria	4,059 (6.6)	1,042 (10.1)	3,017 (5.9)	<0.001

Values are mean ± standard deviation or n (%). HCM, hypertrophic cardiomyopathy; BMI, body mass index; BP, blood pressure; RAS, renin-angiotensin-aldosterone system; BB, beta blocker; CCB, calcium channel blocker; Hb, hemoglobin; eGFR, estimated glomerular filtration rate.

### Incidence of ESRD in the HCM and non-HCM groups

**Figure 1:**
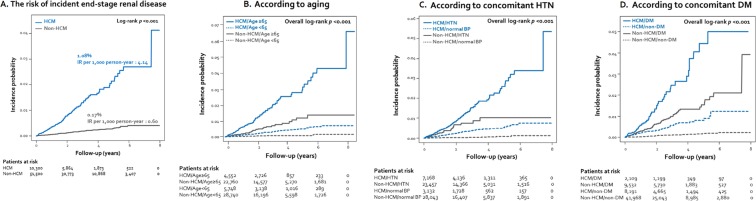
Kaplan Meier curves for the risk of incident ESRD according to HCM and concomitant clinical conditions. Incidence probability stratified by HCM and concomitant clinical conditions **(A–C)** were drawn and compared. Patients with HCM had a higher risk of incident ESRD than those without. This tendency was consistent regardless of aging **(A)**, concomitant hypertension **(B)**, or diabetes mellitus **(C)**. DM, diabetes mellitus; ESRD, end-stage renal disease; HCM, hypertrophic cardiomyopathy; HTN, hypertension.

**Figure 2:**
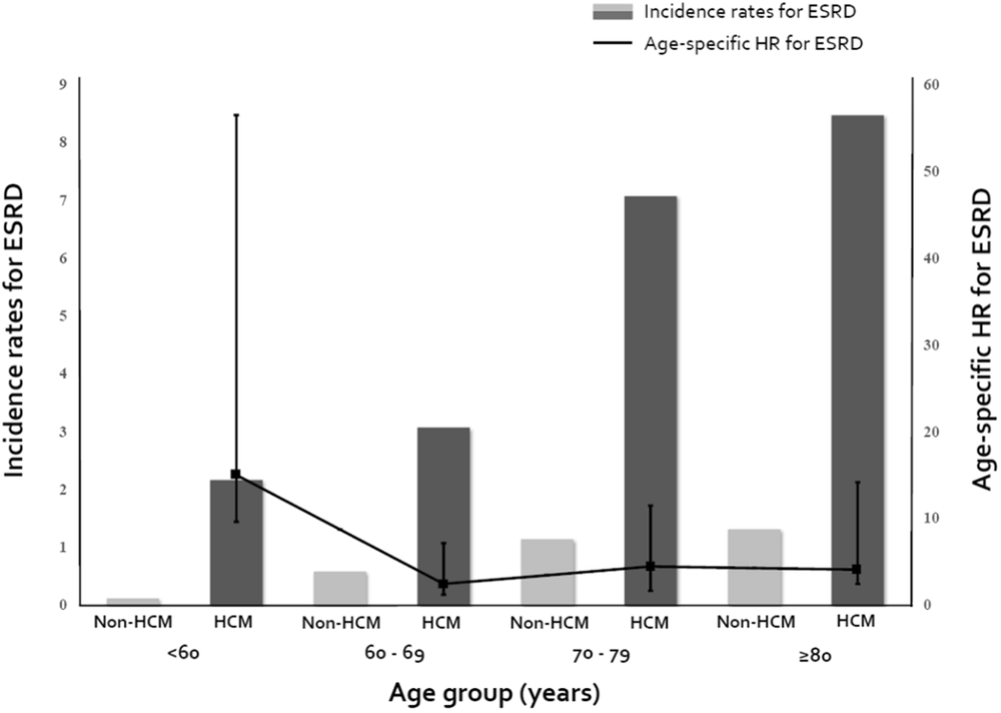
Incidence rates and age-specific risks of incident ESRD. In patients with HCM, the incidence rates for ESRD tended to continuously increase with age, whereas there was an initial peak and subsequent plateau or no significant rise in the age-specific risk for ESRD. ESRD, end-stage renal disease; HCM, hypertrophic cardiomyopathy; HR, hazard ratio.

During follow-up (median 2.8 years, interquartile range 1.5–3.5 months), ESRD was newly diagnosed in 197 subjects (0.32%); 111 in the HCM and 86 in the non-HCM group. ESRD developed more frequently in the HCM than in the non-HCM group (1.08% vs. 0.17%, *p* < 0.001), even though the follow-up duration was longer in the non-HCM group (2.6 vs. 2.8 years, *p* < 0.001). The incidence rates for ESRD were 4.14 and 0.60 per 1,000 person-years in the HCM and the non-HCM groups, respectively (*p* < 0.001) (Table 2). Kaplan-Meier curve demonstrated that HCM patients had a higher incidence probability of ESRD than non-HCM subjects (Fig. 1A). Among ESRD patients, hemodialysis and KT were performed in 103 (92.8%) and 2 (1.8%) cases in the HCM, compared to 82 (95.3%) and 0 (0.0%) cases in the non-HCM group, without a remarkable difference. When stratified by age, in the HCM group, the incidence rates for ESRD tended to gradually increase with age, whereas there was an initial peak and subsequent plateau or no obvious rise in the age-specific risk for ESRD (Table 2, Fig. 2). Particularly, HCM patients under 60 years of age had a 20-fold heightened risk for incident ESRD (age-specific HR 19.88, *p* < 0.001). When analyzed according to sex, the incidence rates for ESRD were higher in the HCM group in both sexes, with similar differences between the HCM and non-HCM groups (Table 2).

**Table 2:** Incidence of ESRD in subjects with vs. without HCM.

	Total (n = 61,800)	HCM group (n = 10,300)	Non-HCM group (n = 51,500)	*p*
ESRD cases, n (%)	197 (0.32)	111 (1.08)	86 (0.17)	<0.001
Follow-up duration, year	2.8 ± 1.6	2.6 ± 1.5	2.8 ± 1.6	<0.001
ESRD incidence (per 1,000 person-years)	1.16	4.14	0.60	<0.001
***by Age group***				
<60	0.41	2.16	0.11	<0.001
60–69	0.97	3.07	0.58	<0.001
70–79	2.05	7.07	1.14	<0.001
≥80	2.40	8.47	1.31	<0.001
***by Sex group***				
Male	1.17	4.05	0.63	<0.001
Female	1.14	4.30	0.56	<0.001

ERSD, end-stage renal disease; other abbreviations as Table 1.

### HCM as an independent predictor for incident ESRD

Age, smoking, concomitant hypertension, DM, hypercholesterolemia, IHD, HF, stroke, AF, and pre-existing renal disease, as well as prior use of anti-platelet agent, RAS blocker, and statin were associated with incident ESRD in the univariate analysis. HCM also demonstrated a strong association with incident ESRD, with an approximately 7-fold increased risk (unadjusted HR 6.90, *p* < 0.001) (Table 3). Notably, the risk of ESRD from HCM was comparable with those from hypertension, DM, and pre-existing renal disease, all of which are widely accepted causes of worsening renal function, emphasizing the unrecognized, but critical hazard of HCM as a contributor to worsening renal function (Table 3, Fig. 1B–D). After adjusting for age and sex [*model 1*], or age, sex, hypertension, DM, pre-existing renal disease and RAS blocker use, which were previously known predictors of ESRD [*model 2*], HCM remained as an independent predictor for ESRD development (adjusted HR 7.04, *p* < 0.001 [*model 1*]; adjusted HR 5.44, *p* < 0.001 [*model 2*]). Furthermore, after adjusting for all variables showing the association in univariate analysis [*model 3*], HCM itself was a robust predictor of ESRD development (adjusted HR 3.93, *p* < 0.001) (Table 3). Sensitivity analysis was performed to demonstrate the direct association between HCM itself and ESRD development, regardless of pre-existing renal disease (Table 4A) and heart failure (Table 4B), and similar results were obtained.

**Table 3:** The univariate and multivariate analysis of the risk of ESRD.

Univariate analysis		
Variables	Unadjusted HR (95% CI)	p
Age (per 10 years increment)	1.82 (1.58–2.09)	<0.001
Male sex	1.03 (0.77–1.39)	0.834
Smoking	0.51 (0.33–0.78)	0.002
BMI ≥ 25 kg/m^2^	0.91 (068–1.22)	0.525
Income lower 20%	0.95 (0.60–1.51)	0.824
Hypertension	5.55 (3.77–8.19)	<0.001
Diabetes mellitus	4.47 (3.38–5.93)	<0.001
Hypercholesterolemia	2.18 (1.65–2.89)	<0.001
Ischemic heart disease	3.60 (2.72–4.77)	<0.001
Heart failure	5.94 (4.38–8.05)	<0.001
Stroke	2.52 (1.73–3.68)	<0.001
Atrial fibrillation	3.45 (2.60–4.58)	<0.001
Pre-existing renal disease	6.14 (4.32–8.72)	<0.001
Prior use of anti-platelets	3.72 (2.81–4.93)	<0.001
Prior use of RAS blocker	4.93 (3.65–6.65)	<0.001
Prior use of statin	2.85 (2.15–3,77)	<0.001
HCM	6.90 (5.21–9.15)	<0.001
**Multivariate analysis**		
**Models**	**Adjusted HR of HCM (95% CI)**	**p**
Model 1^*^	7.04 (5.31–9.33)	<0.001
Model 2^†^	5.44 (4.08–7.24)	<0.001
Model 3^‡^	3.93 (2.82–5.46)	<0.001

Multivariate models were adjusted for age and sex^*^ and age, sex, hypertension, diabetes mellitus, predisposing renal disease, and use of RAS blocker^**†**^, and age, sex, smoking, hypertension, diabetes mellitus, hypercholesterolemia, ischemic heart disease, heart failure, stroke, atrial fibrillation, pre-existing renal disease, and prior use of anti-platelet, RAS blocker, and statin^‡^, respectively. HR, hazard ratio; CI, confidence interval; other abbreviations as Tables 1 and 2.

**Table 4:** Sensitivity analysis of the risk of ESRD by HCM according to pre-existing renal disease (A) and heart failure (B).

*Univariate analysis*		
Variables	Unadjusted HR (95% CI)	*p*
**A-1. In subjects without pre-existing renal disease (n** = **50,722)**		
HCM	8.19 (5.64–11.88)	<0.001
***Multivariate analysis***		
**Models**	**Adjusted HR of HCM (95% CI)**	***p***
Model 1*	8.46 (5.83–12.28)	<0.001
Model 2^†^	6.98 (4.76–10.23)	<0.001
Model 3^‡^	5.83 (3.79–8.98)	<0.001
**A-2. In subjects with pre-existing renal disease (n** = **11,078)**		
HCM	4.73 (3.07–7.30)	<0.001
Model 1*	4.69 (3.04–7.25)	<0.001
Model 2^†^	4.06 (2.62–6.28)	<0.001
Model 3^‡^	2.97 (1.81–4.89)	<0.001
**B-1. In subjects without heart failure (n** = **59,986)**		
HCM	6.16 (4.47–8.33)	<0.001
Model 1*	6.33 (5.74–8.64)	<0.001
Model 2^†^	4.84 (3.53–6.64)	<0.001
Model 3^‡^	4.38 (3.08–6.23)	<0.001
**B-2. In subjects with heart failure**^**§**^ **(n** = **1,814)**		
HCM	2.43 (1.11–5.30)	<0.001
Model 1*	3.00 (1.36–6.64)	<0.001
Model 2^†^	3.02 (1.36–6.68)	<0.001
Model 3^‡^	3.31 (1.46–7.53)	<0.001

Multivariate models were adjusted for age and sex^*^ and age, sex, hypertension, diabetes mellitus, and use of RAS blocker^**†**^, and age, sex, smoking, hypertension, diabetes mellitus, hypercholesterolemia, ischemic heart disease, heart failure, stroke, atrial fibrillation, and prior use of anti-platelet, RAS blocker, and statin^‡^, respectively. Patients with heart failure^§^ was defined as the diagnostic code (I50) with a history of admission for HF. All abbreviations as Tables 1–3.

### Subgroup analysis of the impact of HCM on developing ESRD

**Figure 3:**
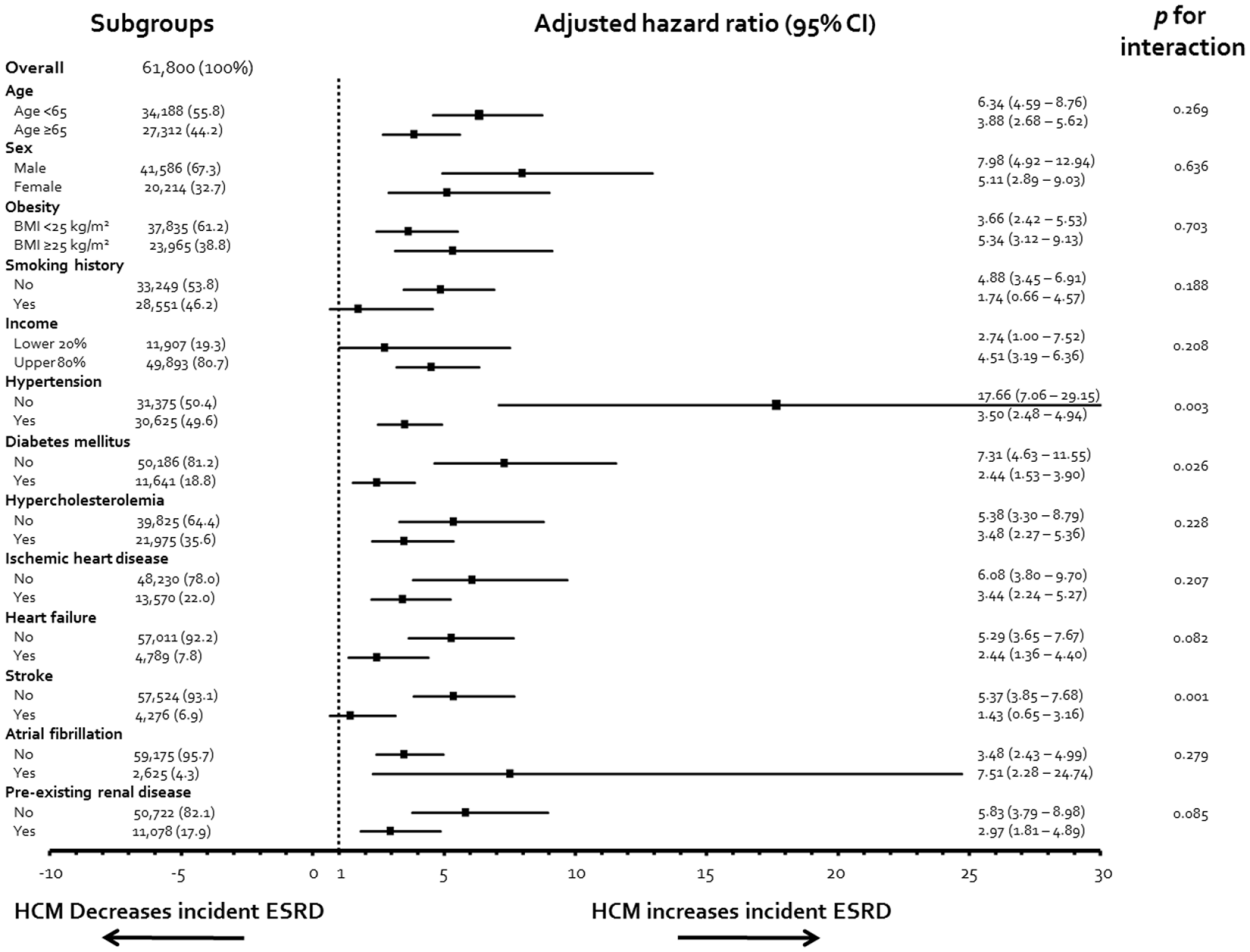
Adjusted HR of each risk factors for the development of ESRD. HCM consistently increased the risk of ESRD, regardless of age, sex, lifestyle behavior, and associated medical illnesses, with a HR over 1.0 in all subgroups. Note that subgroups that were deemed low risk for cardiovascular morbidity or mortality, i.e., younger than 65 years, non-smoker, no history of comorbidities such as hypertension, diabetes mellitus, heart failure or stroke, demonstrated a high adjusted HR for ESRD development. Also note that HCM patients with atrial fibrillation had a higher risk of incident ESRD than those without, although this comparison did not reach statistical significance. ESRD, end-stage renal disease; HCM, hypertrophic cardiomyopathy; HR, hazard ratio.

In general, HCM showed a consistent tendency to increase the risk of ESRD, independent of age, sex, lifestyle behaviors, and concomitant medical illnesses, with a HR > 1.0 in all subgroups. Of note, subgroups that were deemed low risk for cardiovascular morbidity or mortality, i.e., younger than 65 years, non-smoker, no history of comorbidities such as hypertension, DM, HF, or stroke, demonstrated a high adjusted HR for ESRD development (Fig. 3). This subgroup analysis also indicates the impact of HCM itself on developing ESRD, free from the associated clinical conditions.

## Discussion

The main findings of the current study can be summarized as follows: (1) the incidence of ESRD is higher in HCM patients than non-HCM subjects in all age groups and in both sexes; (2) HCM is an independent predictor of ESRD, regardless of generally-accepted prognosticators such as age, hypertension, DM, pre-existing renal disease, or prior use of RAS blocker; and (3) especially, in younger and healthier men, the risk of developing ESRD by HCM tended to increase. Taken together, this large-scale nationwide cohort study firstly demonstrated the heightened risk of ESRD in HCM patients, introduced HCM as a novel risk factor for ESRD, and suggested that early and active surveillance for renal function is potentially helpful for improving the prognosis and quality of life in HCM patients.

ESRD patients have increased in recent decades due to a rapidly aging society and increasing prevalence of the causative comorbidities^10^. The Korean Society of Nephrology reported that the incidence of ESRD was approximately 0.32 per 1,000 person-years in the general population, and doubled in adults aged ≥65 years^11,12^. In this epidemiologic data, ESRD was detected in 197 patients of the total cohort (0.32%); 111 (1.08%) in the HCM, and 86 (0.17%) in the non-HCM, during a median 2.8 years of follow-up. Although the absolute incidence of ESRD in HCM may not seem high, its impact on clinical practice and prognosis may be considerable because of critical complications and high socioeconomic burden of both diseases^4,13,14^. The incidence of ESRD in the non-HCM group was 0.60 per 1,000 person-years, similar to the known incidence in the general population reported in other studies, implying a well-represented study population^10–12,15^. Patients with newly diagnosed ESRD in the HCM was approximately 7-fold higher than those in the non-HCM (4.14 vs. 0.60 per 1,000 person-year, *p* < 0.001). The difference in ESRD incidence between 2 groups was sustained, irrespective of age and sex. Surprisingly, the high incidence rate of ESRD in HCM patients was comparable to that from diabetic nephropathy or hypertensive nephrosclerosis, two major causes of ESRD^10^. These results indicate that HCM, a previously unrecognized, but novel risk factor of renal dysfunction, can contribute to ESRD development. Therefore, early risk stratification and active surveillance for renal function need to be considered in HCM patients, to preserve renal function, guide the clinical practice, and potentially improve their long-term prognosis and quality of life.

To date, numerous studies have demonstrated that ESRD can occur as a complication of cardiac diseases^16–19^; however, its relationship with HCM has not fully elucidated. Several studies have propounded that HCM can worsen renal function^20,21^. In an observational study of 10 pediatric patients with chronic dialysis, four of them showed asymmetric septal hypertrophy on echocardiography, and two also had signs suggestive of LV outflow tract (LVOT) obstruction^20^. More recently, in a small retrospective cross-sectional study, Wang *et al*. noted that HCM patients had lower eGFR and higher urine 8-hydroxy-2-deoxyguanosine levels, surrogate markers of impaired renal function^21^. However, previous reports only provided indirect evidence of potential association between HCM and renal dysfunction in limited cases, and could not establish a temporal relationship. In this large-scale longitudinal study, we proved that HCM was a strong predictor of ESRD development, even after adjusting for other well-known confounders. Additional sensitivity analysis verified that a higher risk of ESRD development in HCM was not a simple consequence mediated by pre-existing renal disease. Furthermore, we observed that the younger and healthier HCM subgroup was prone to develop ESRD, again implying a direct impact of HCM on renal dysfunction.

Although the pathogenesis of ESRD in HCM is likely multifactorial, we assumed that renal dysfunction may be attributed mainly to LV diastolic dysfunction. Pronounced LV hypertrophy leads to reduced LV cavity size, a shift in the pressure-volume relationship, resulting in a steeper rise in intra-cavitary pressure for a given increase in LV filling volume. Theoretically, a stiff LV induced by diastolic dysfunction in HCM can increase LV filling pressure and renal venous pressure through RAS activation, facilitate an unfavorable volume distribution, and consequently disturb renal function^22^. It has been already shown that renal impairment was associated with LV diastolic dysfunction under various cardiovascular diseases, rather than systolic dysfunction or hypertrophied LV *per se*^23–26^. In a study of 289 hypertensive patients, renal function deteriorated in parallel with the progression of LV diastolic dysfunction^26^. Another hypothesis is chronic renal hypoperfusion in cases of LVOT obstruction, which occurs in approximately 25% of HCM patients^27^. This hypothesis can be supported by the concept of cardiorenal syndrome type 2, characterized by chronic abnormalities in cardiac function causing progressive renal dysfunction^28^. Hemodynamic instability from LVOT obstruction can cause chronic and repetitive renal ischemic injury, resulting in a chronic deterioration of renal function. This has been advocated by a case report from Japanese researchers, wherein a 70-year-old female HCM patient completely recovered from renal dysfunction following resolution of LVOT obstruction after alcohol septal myocardial ablation^29^. Considering that AF is the most common arrhythmia in HCM^5^, it is likely that renal infarction as peripheral thromboembolism related to AF may contribute to a substantial proportion of cases with renal dysfunction in HCM. In one study conducted in Israel over 18 years, renal infarction was detected in 44 patients with AF; 42% showed renal dysfunction, of which 3 cases progressed to ESRD^30^.

There are several limitations in this study. First, this is a large-scale cohort study using claims data, with all definitions of diseases based on diagnostic codes. We could not directly analyze echocardiographic or cardiac magnetic resonance results of the patients enrolled owing to the innate limitation of claims data. We could not directly analyze echocardiographic or cardiac magnetic resonance results of the patients enrolled owing to the innate limitation of claims data. Therefore, the small possibility of misdiagnosis and omission cannot be excluded. We could not demonstrate data regarding the morphological phenotype of HCM and the presence or absence of LVOT obstruction, either. However, the final diagnosis of HCM and ESRD was based on clinical and/or imaging evidences validated by external medical experts and health insurance professionals. Besides, great efforts were made to minimize errors by refining and validating the definitions of variables^31–33^. Furthermore, we included only HCM patients registered in the National Health Insurance Service (NHIS) *Rare Intractable Diseases* program to increase diagnostic reliability. In a validation study by our institution (n = 1,110), HCM definition combining ICD-10 codes and the diagnostic code from *Rare Intractable Diseases* showed a high positive predictive value, also reinforcing the reliability of our definitions^31^. Second, despite being the first epidemiologic study to demonstrate the link between renal dysfunction and HCM, the pathophysiology underlying this association could not be fully evaluated. Thus, further prospective studies are warranted. Third, because the follow-up duration was relatively short (a median duration of 2.8 years), it might be possible that HCM patients had a constellation of risk factors for ESRD development more than non-HCM counterpart did. In addition, the pathophysiology to clearly explain the link between HCM and ESRD could not be evaluated under the observational study design. Further long-term prospective studies are mandatory. Finally, we could not exclude the presence of currently unknown confounders that may worsen renal function. Nevertheless, this study has consistently demonstrated that HCM increased the risk of ESRD in three different multivariable models and subgroup analysis, where potential confounders were comprehensively adjusted for. Therefore, we could infer the strong association between HCM and ESRD. In conclusion, this large-scale nationwide population-based cohort study firstly demonstrated that HCM increased the risk of ESRD development, regardless of other known prognosticators. Thus, early and active surveillance for renal function can be helpful to improve the prognosis and quality of life of HCM patients in the contemporary management strategy era for this genetic disease with low mortality.

## Methods

### Data source and study population

**Figure 4:**
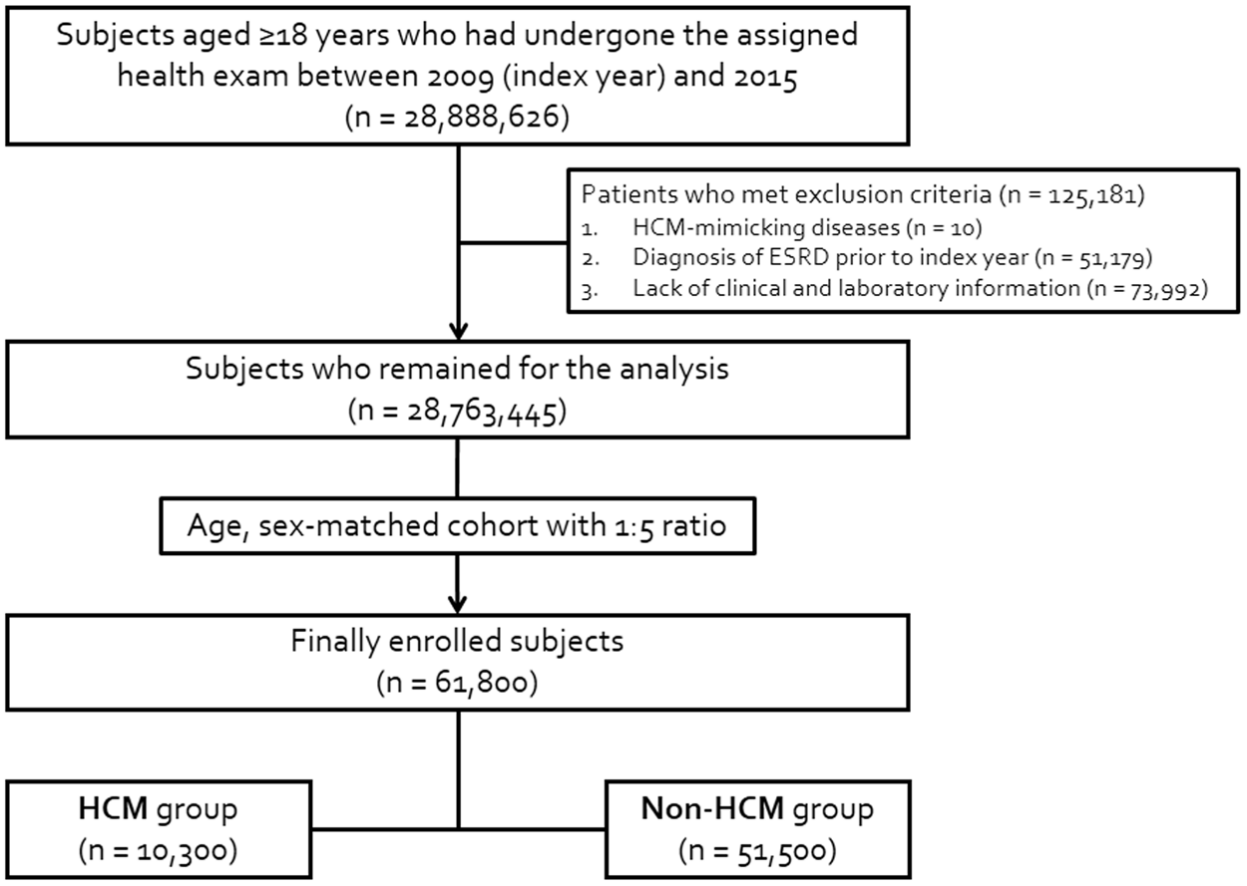
Schematic flow for study population enrollment. ESRD, end-stage renal disease; HCM, hypertrophic cardiomyopathy.

The claims data of the NHIS of Korea was used. The NHIS is a mandatory universal health insurance program started in 1963, managed by the government since 1989 and incorporated into a single database with the Medical Aid Program for low-income bracket since 2006, offering the medical information of the entire Korean population^34^. All the insured are meant to undergo a standardized national health examination biennially. Additionally, the Health Insurance Review and Assessment Service (HIRA) provides a regular evaluation, quality control, and feedback about the whole medical care in Korea. Through NHIS and HIRA linkage, the big database including medical information of all Koreans was established under the strict supervision of the Ministry of Health and Welfare. This database encompasses demographics, medical facility utilization history, diagnoses, prescriptions, and the national health exam results of a given year^35^. From this database, we collected subjects aged ≥18 years undergoing the assigned health exam between 1 January, 2009 and 31 December, 2015 (n = 28,888,626). After excluding 10 patients with HCM-mimicking diseases, including 4 Fabry disease (E75.2, V117) and 6 amyloidosis (E85, V121), 51,179 previously diagnosed with ESRD and 73,992 with missing variables (n = 28,763,445), we recruited 10,300 patients with HCM and matched with 51,500 age- and sex-matched controls (1:5 ratio) for the final analysis (Fig. 4). The study protocol conforms to the ethical guidelines of the 1975 Declaration of Helsinki, and informed consent was waived as we used the anonymized and unidentified data. This was approved by the Institutional Review Board of our institution.

### Definition of HCM and its validity

HCM was defined using the International Classification of Disease, 10^th^ Revision (ICD-10) codes (I42.1–42.2). In Korea, HCM falls under the *Rare Intractable Diseases* category, where patients are designated as special medical aid beneficiaries with expanding benefit of the NHIS. Since 2006, the government has introduced an initiative covering 90% of all medical expenses claimed by these patients. Therefore, the diagnosis of HCM is strictly determined and monitored by thorough verification with clinical and imaging evidences, and periodical reviews by medical experts and health insurance professionals, according to an act established by the Ministry of Health and Welfare. Furthermore, the definition of HCM by the diagnostic code was validated in the previous study by our institution, by reviewing medical records including echocardiography or cardiac magnetic resonance imaging and comparing the diagnostic accuracy^31^. Therefore, data for HCM in this study is considered reliable.

### Clinical and laboratory evaluation

Detailed methods previously published were used for this study^31–33^. Age and sex data was retrieved from the resident registration number generated at birth registration. Income level was dichotomized at the lowest 20%. Anthropometric information, including height, weight, and blood pressure, was collected and documented by a trained nurse on the day of the assigned health exam. Body mass index was estimated as weight divided by height in meters squared (kg/m^2^). A self-reported questionnaire was used to assess current smoking status, defined as current consumption of at least 1 cigarette a day. Comorbidities were defined using the ICD-10 codes and the prescription lists from the NHIS database. Hypertension was defined as a previous diagnosis of hypertension (I10-I11) and use of antihypertensive drugs. DM was defined as a previous DM diagnosis (E10-14) or use of 1 or more oral hypoglycemic agents or insulin. Hypercholesterolemia was defined as a diagnosis of hypercholesterolemia (E78) or use of lipid-lowering agents. Pre-existing renal disease was defined as a diagnosis of renal parenchymal, vascular, and ureteral diseases confirmed by nephrologists before the index year (N00-19, N25). IHD, previous stroke (including transient ischemia attack), HF, and AF were identified using the ICD-10 codes (I20-25 for IHD; I60-69 or G45.8-9 for stroke; I50 for HF; and I48 for AF). Among the prescription lists, information regarding renin-angiotensin-aldosterone system blockers, beta blockers, calcium-channel blockers, anti-platelet agents, and statins which were licensed in Korea during the follow-up were separately collected. All laboratory evaluations were performed in certified hospitals that were subjected to periodic quality control by the NHIS. It included hemoglobin, fasting glucose, serum total cholesterol, triglycerides, high-density lipoprotein cholesterol, low-density lipoprotein cholesterol, and eGFR level from the health exam results. Urine protein was measured semi-quantitatively by dipstick on clean, midstream urine, and reported in the following grades: absent, trace (±), 1+ to 4+, corresponding to urine protein concentrations of undetectable, 10 mg/dL, 30 mg/dL, 100 mg/dL, 300 mg/dL, and 1 g/dL, respectively. Based on this result, proteinuria was defined as a grade of 1+ or greater.

### Study endpoint and follow-up

The study population was followed up to the date of ESRD diagnosis or until December 31, 2016, whichever came first. The study outcome was an incident ESRD during follow-up. ESRD was confirmed by ICD-10 codes (N18-19, Z49, Z94.0, Z99.2) with estimated glomerular filtration rates (eGFR) <15 mL/min/1.73 m^2^ or the initiation and maintenance for >3 months of renal replacement therapy and/or kidney transplantation (KT) (R3280 [KT], O7011-7020, V001 [hemodialysis], O7071-7075, V003 [peritoneal dialysis])^36,37^. In Korea, ESRD falls under the *Rare Intractable Diseases* category; thus, 90% of the expenses for medical care and dialysis is reimbursed by the NHIS. Similar to HCM, ESRD is strictly determined by reviewing patients’ medical history and confirmed by medical experts and health insurance professionals. All ESRD patients and related procedures, including dialysis and KT, are registered as special medical aid beneficiaries, and supervised by the NHIS. The validity of ESRD definition by the diagnostic code from *Rare Intractable Diseases* was also evaluated in the nationwide population-based study in Korea^32^. Hence, data regarding ESRD, as confirmed by the NHIS, is trustworthy.

### Statistical analysis

Statistical analyses were conducted with SAS version 9.3 (SAS Institute, Cary, NC, USA). Categorical variables (frequencies and percentages) were compared using the χ^2^ test or Fisher’s exact test, and continuous variables (mean ± standard deviation) were analyzed by the Student’s *t-*test or Wilcoxon rank sum test for independent samples. The incidence rates of ESRD were calculated by dividing the number of detected cases by follow-up duration, and were expressed as per 1,000 person-years in the total cohort and the stratified subgroups according to age and sex, respectively. The incidence probability was displayed between subjects with HCM and non-HCM by Kaplan-Meier analysis with the log-rank test. To estimate the risk and determine the predictors of ESRD, Cox regression analyses were used. The risk of ESRD was expressed as a hazard ratio (HR) and corresponding 95% confidence interval (CI) from univariate and multivariate analyses with forward selection in order. Sequential Cox analyses using 3 nested models were performed to evaluate the independent predictive value of HCM for ESRD. We conducted the sensitivity analyses by separating HCM patients with pre-existing renal disease from those without, as pre-existing renal disease might affect ESRD development later in life.

## Data Availability

The data is unavailable outside NHIS system.
